# Supplementary material for: Inconsistent shifts in warming and temperature variability are linked to reduced avian fitness
Source: Nat Commun. 2023 Nov 16;14:7400. doi: 10.1038/s41467-023-43071-y (PMC10654519; doi:10.1038/s41467-023-43071-y)
Supplement: Supplementary file 1 — Supplementary Information [file 41467_2023_43071_MOESM1_ESM.pdf]

SUPPLEMENTAL TABLES AND FIGURES

For: Inconsistent shifts in warming and temperature variability are linked to reduced avian fitness

Conor C Taff & J. Ryan Shipley

Table S1: Total number of breeding records at incubation stage and provisioning stage for each included species.

Common name	Scientific name	Incubation Records	Provisioning Records
American Robin	*Turdus migratorius*	2101	1620
Barn Swallow	*Hirundo rustica*	1003	896
Bewick's Wren	*Thryomanes bewickii*	1421	1242
Black-Capped Chickadee	*Poecile atricapillus*	2993	2348
Black-Crested Titmouse	*Baeolophus atricristatus*	527	468
Brown-Headed Nuthatch	*Sitta pusilla*	375	334
Carolina Chickadee	*Poecile carolinensis*	5287	4411
Chestnut-Backed Chickadee	*Poecile rufescens*	951	816
Eastern Bluebird	*Sialia sialis*	80447	68534
Eastern Phoebe	*Sayornis phoebe*	998	877
House Finch	*Haemorphous mexicanus*	627	517
House Wren	*Troglodytes aedon*	16946	14121
Mountain Bluebird	*Sialia currocoides*	9051	7897
Mountain Chickadee	*Poecile gambeli*	526	457
Mourning Dove	*Zenaida macroura*	524	404
Northern Cardinal	*Cardinalis cardinalis*	512	384
Oak Tit	*Baeolophus inornatus*	1066	937
Prothonotary Warbler	*Protonotaria citrea*	2121	1907
Purple Martin	*Progne subis*	85368	77828
Tree Swallow	*Tachycineta bicolor*	64222	55338
Tufted Titmouse	*Baeolophus bicolor*	1224	1006
Violet-Green Swallow	*Tachycineta thalassina*	2410	2078
Western Bluebird	*Sialia mexicana*	20335	18273
White-Breasted Nuthatch	*Sitta carolinensis*	479	437

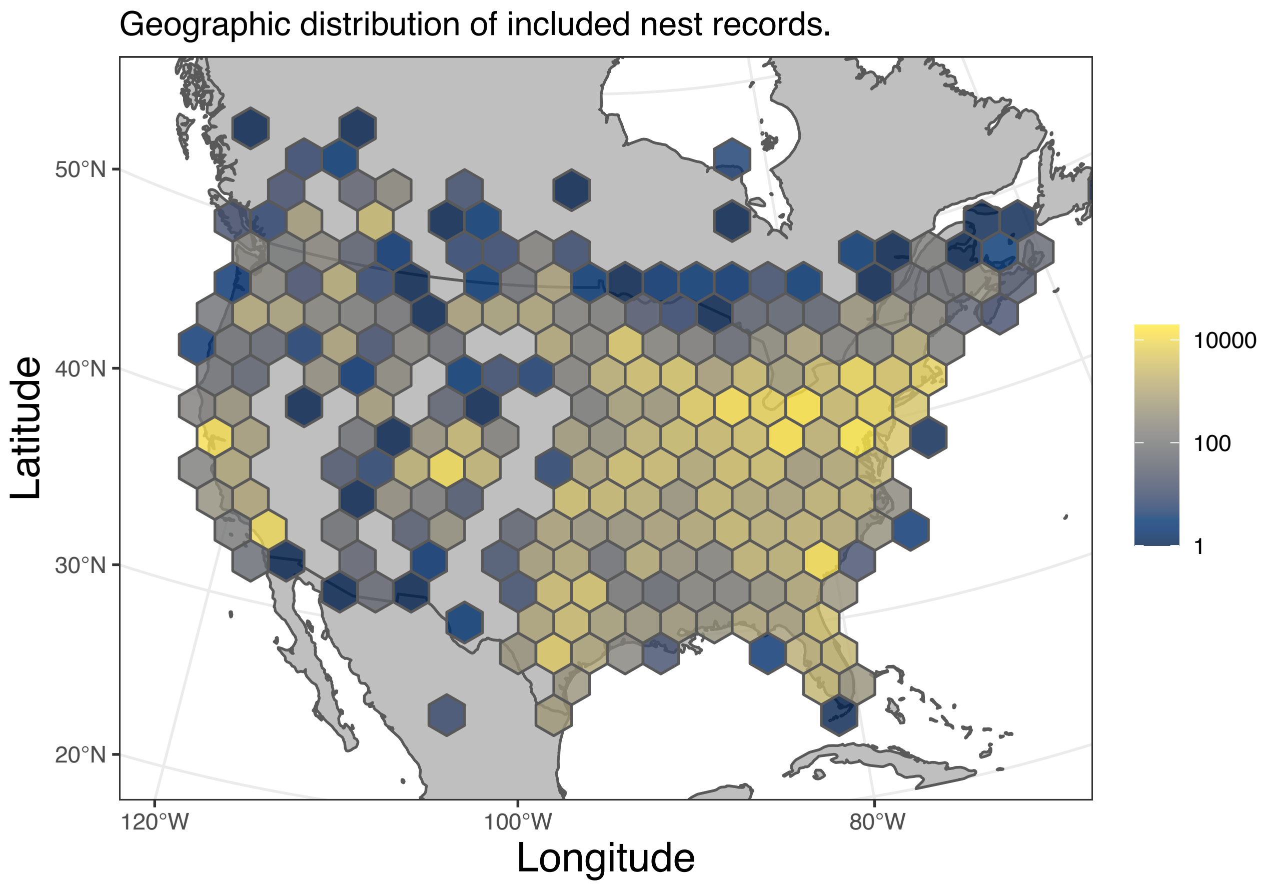

Figure S1: Number of breeding records included within each grid cell for all species and years from the combined datasets after filtering. A total of 301,514 records from 24 species are included from 1995 to 2020.

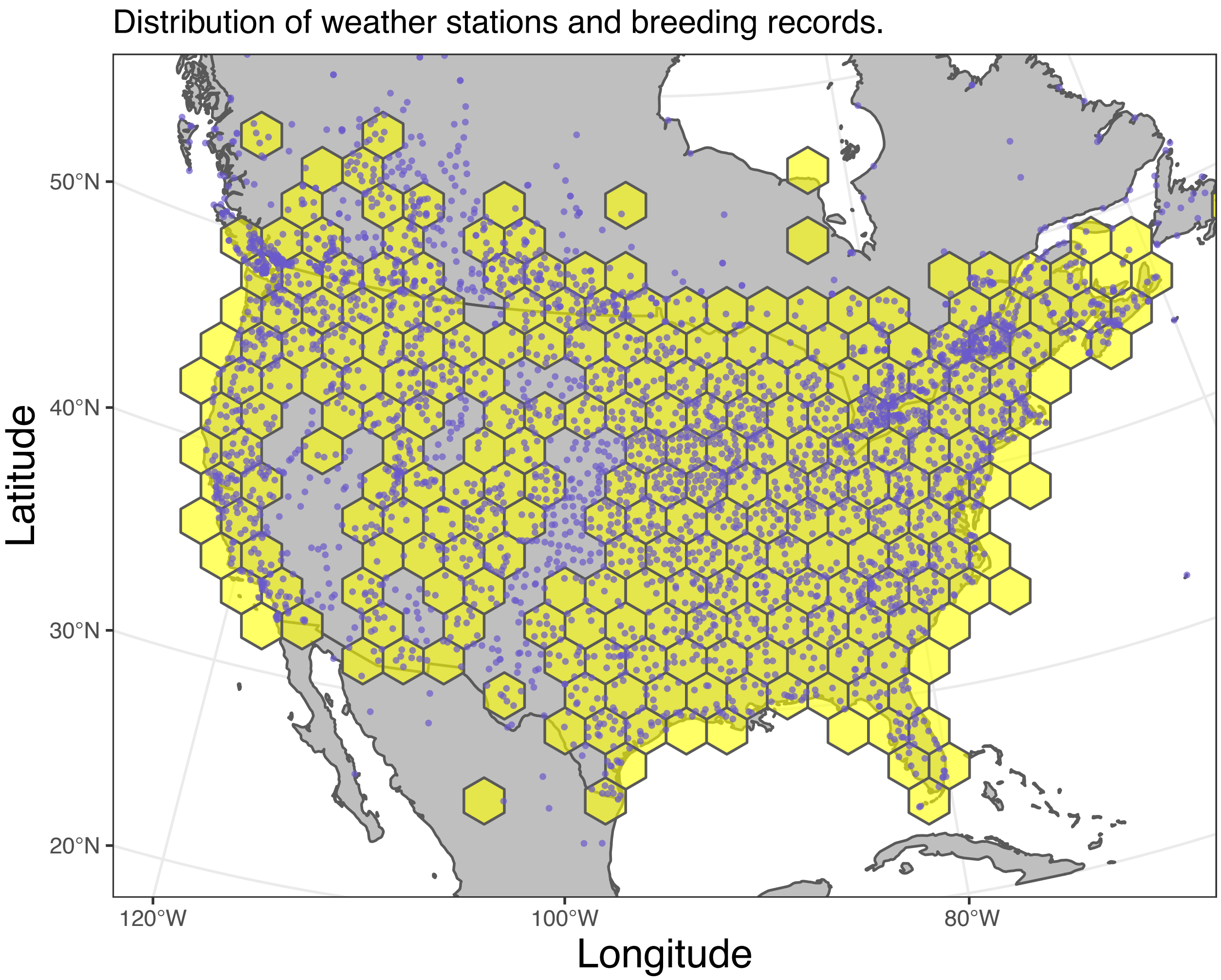

Figure S2: Weather stations used to access historical temperature data. Individual points show station locations with data downloaded from NOAA (n = 2608) or Environment and Climate Change Canada (n = 1125). Stations that had at least 50 years of available data were included. Yellow hexagons show the spatial extent of the avian breeding records used in the analysis to illustrate how coverage of temperature data corresponds to the main dataset.

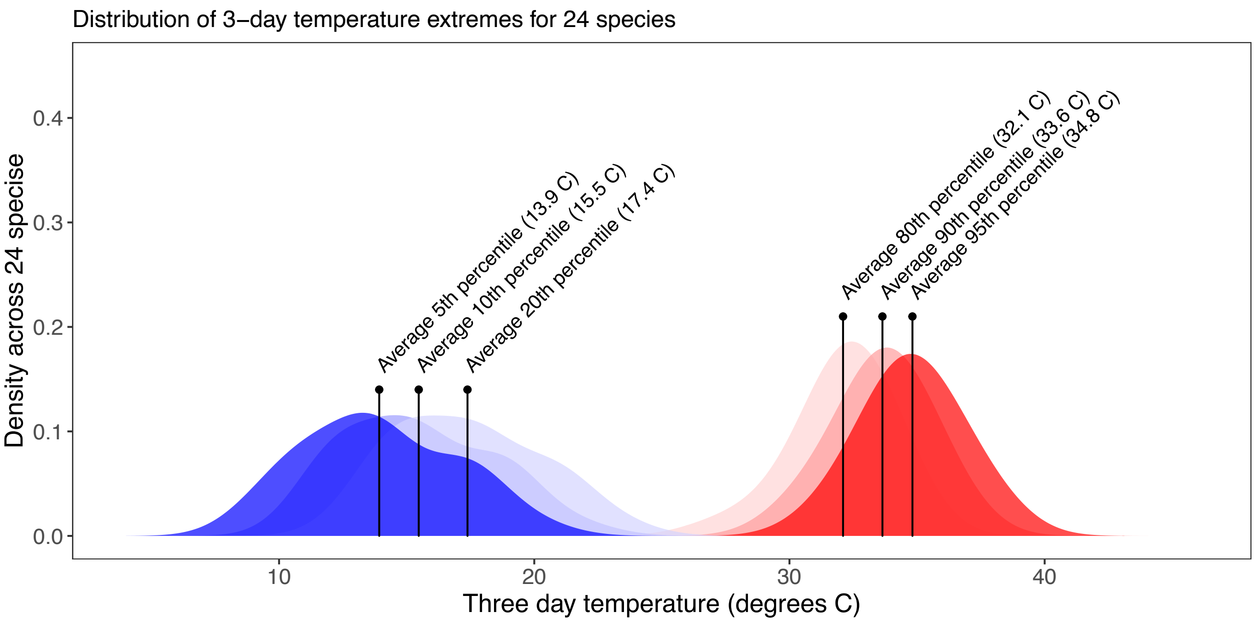

Figure S3: Distribution of extreme climatic events for the 24 species included in this study used to determine threshold temperatures for analysis of historical cold snap and heatwave timing. Six distrubutions are shown, illustrating the 5th, 10th, and 20th percentile of the coldest three day period during breeding attempts and the 80th, 90th, and 95th percentile of hottest three day period during breeding attempts. Black lines indicate the raw averages across 24 species for each distribution and these values were used in the historical analysis described in the text.

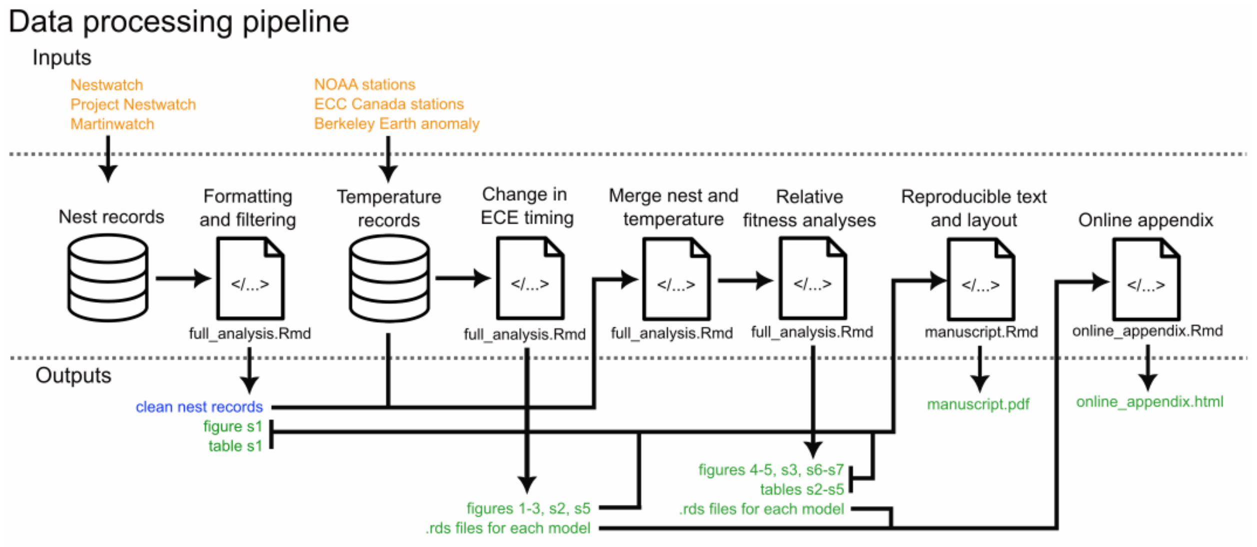

Figure S4: Schematic illustration of the data analysis workflow from raw data inputs to data outputs, manuscript file, and online supplement. To reproduce the analyses, raw data shown in orange will need to be accessed from each appropriate repository in order to produce the output shown in blue. Downstream output files shown in green are available in the archived data and code repository along with all of the scripts referenced in the figure.

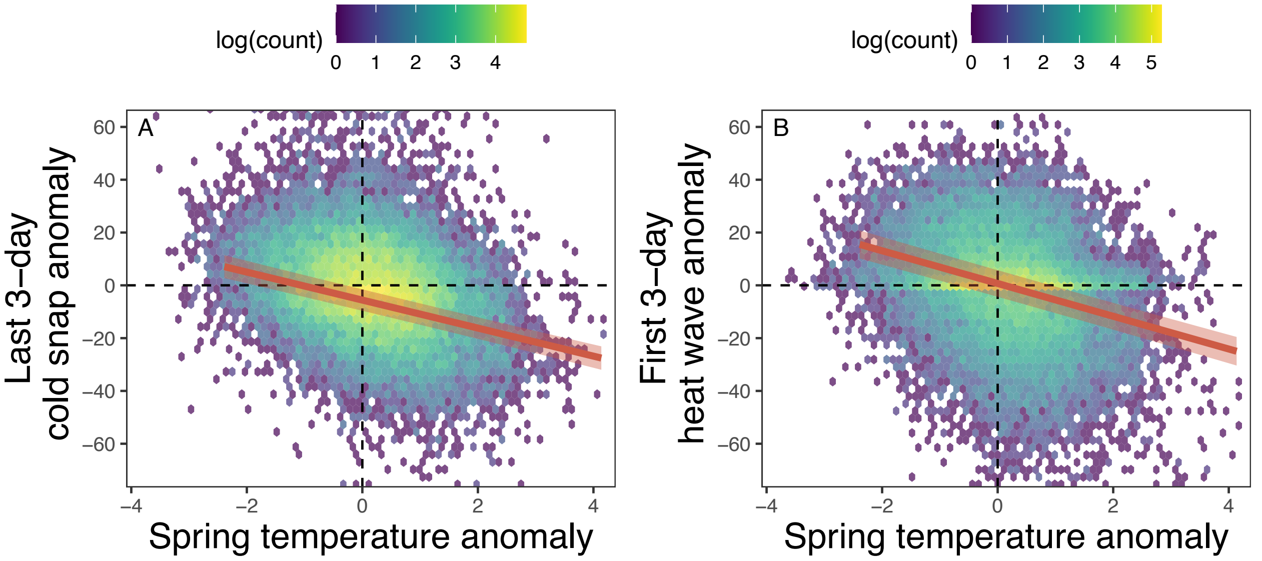

Figure S5: Spring average temperature anomaly in relation to the date of the last three day cold snap (A) or earliest heatwave (B). Grid shows the count of total cell level observations at each combination with all years counted. Red line is a fit from a GAM that includes a basis spline for year and tensor smooth for latitude and longitude to account for spatial and temporal autocorrelation. Shaded red region shows the 95% confidence interval of the fit GAM.

Table S2: Point estimates for the expected relative fitness of each species during a two standard deviation cold snap or heatwave based on GAMs described in text. Numbers in brackets show 95-percent confidence intervals.

	Incubation cold snap	Incubation heatwave	Provisioning cold snap	Provisioning heatwave
American robin	0.96 [0.80, 1.12]	1.02 [0.89, 1.14]	0.75 [0.65, 0.85]	0.90 [0.79, 1.01]
Barn swallow	1.10 [0.92, 1.28]	1.08 [0.94, 1.22]	0.98 [0.85, 1.10]	0.95 [0.86, 1.05]
Bewick's wren	0.98 [0.85, 1.11]	0.93 [0.78, 1.07]	1.00 [0.90, 1.10]	0.95 [0.86, 1.03]
Black-capped chickadee	1.02 [0.93, 1.11]	0.96 [0.82, 1.09]	0.90 [0.84, 0.95]	0.84 [0.77, 0.91]
Black-crested titmouse	1.02 [0.79, 1.26]	0.95 [0.74, 1.15]	0.86 [0.75, 0.97]	1.01 [0.92, 1.11]
Brown-headed nuthatch	0.89 [0.71, 1.07]	1.00 [0.78, 1.21]	0.87 [0.73, 1.01]	0.75 [0.60, 0.90]
Carolina chickadee	0.92 [0.85, 0.99]	0.99 [0.92, 1.06]	0.94 [0.90, 0.98]	0.94 [0.88, 1.01]
Chestnut-backed chickadee	0.95 [0.73, 1.18]	0.99 [0.87, 1.11]	0.94 [0.85, 1.03]	0.88 [0.78, 0.98]
Eastern bluebird	0.93 [0.90, 0.95]	0.86 [0.81, 0.91]	0.87 [0.86, 0.89]	0.87 [0.85, 0.90]
Eastern phoebe	1.06 [0.86, 1.26]	1.02 [0.89, 1.15]	0.74 [0.62, 0.86]	0.85 [0.76, 0.94]
House finch	1.11 [0.89, 1.33]	1.01 [0.77, 1.25]	0.85 [0.69, 1.00]	0.80 [0.66, 0.93]
House wren	0.97 [0.92, 1.02]	1.00 [0.95, 1.06]	0.91 [0.89, 0.94]	0.93 [0.89, 0.96]
Mountain bluebird	0.80 [0.72, 0.88]	0.91 [0.81, 1.01]	0.85 [0.79, 0.90]	0.89 [0.83, 0.96]
Mountain chickadee	0.64 [0.38, 0.91]	0.91 [0.73, 1.10]	0.89 [0.76, 1.03]	0.91 [0.71, 1.11]
Mourning dove	0.96 [0.63, 1.29]	0.83 [0.56, 1.10]	0.92 [0.73, 1.11]	0.92 [0.76, 1.09]
Northern cardinal	0.96 [0.55, 1.37]	1.15 [0.79, 1.51]	0.80 [0.49, 1.10]	0.81 [0.58, 1.04]
Oak titmouse	1.01 [0.89, 1.13]	1.08 [0.97, 1.19]	0.93 [0.84, 1.03]	1.04 [0.90, 1.19]
Prothonotary warbler	0.79 [0.64, 0.94]	1.00 [0.90, 1.11]	0.99 [0.92, 1.05]	1.00 [0.93, 1.06]
Purple martin	0.86 [0.83, 0.89]	0.98 [0.95, 1.00]	0.87 [0.85, 0.89]	0.94 [0.92, 0.96]
Tree swallow	0.85 [0.82, 0.89]	0.98 [0.95, 1.01]	0.91 [0.89, 0.93]	0.93 [0.91, 0.95]
Tufted titmouse	0.91 [0.77, 1.05]	0.91 [0.78, 1.04]	0.97 [0.88, 1.07]	0.93 [0.84, 1.02]
Violet-green swallow	1.03 [0.92, 1.14]	1.03 [0.92, 1.13]	0.83 [0.74, 0.92]	0.92 [0.83, 1.01]
Western bluebird	0.84 [0.78, 0.90]	0.98 [0.93, 1.03]	0.79 [0.74, 0.85]	0.92 [0.88, 0.97]
White-breasted nuthatch	0.79 [0.48, 1.10]	0.94 [0.76, 1.13]	0.75 [0.60, 0.90]	0.96 [0.69, 1.23]

Table S3: Point estimates for the expected relative fitness of eastern bluebirds during a two standard deviation cold snap or heatwave based on GAMs described in text. Breeding records are split into five latitude bands with an equal number of records per band; the southernmost band includes nests below 33.5 degrees latitude and is followed by 33.5 to 38.1 degrees, 38.1 to 40.0 degrees, 40.0 to 41.7 degrees, and finally nests above 41.7 degrees in the northernmost band. Numbers in brackets show 95-percent confidence intervals.

	Incubation cold snap	Incubation heatwave	Provisioning cold snap	Provisioning heatwave
Northernmost	0.91 [0.85, 0.96]	1.03 [0.98, 1.08]	0.87 [0.83, 0.91]	0.89 [0.83, 0.96]
North	0.98 [0.94, 1.03]	0.91 [0.84, 0.99]	0.90 [0.87, 0.94]	0.94 [0.89, 0.98]
Middle	0.97 [0.92, 1.01]	0.94 [0.89, 1.00]	0.98 [0.95, 1.02]	0.95 [0.90, 1.00]
South	0.99 [0.94, 1.04]	0.96 [0.90, 1.01]	1.01 [0.98, 1.04]	0.96 [0.92, 1.00]
Southernmost	0.97 [0.91, 1.02]	0.84 [0.76, 0.92]	1.00 [0.96, 1.03]	0.90 [0.85, 0.95]

Purple martin sensitivity to cold snaps and heatwaves

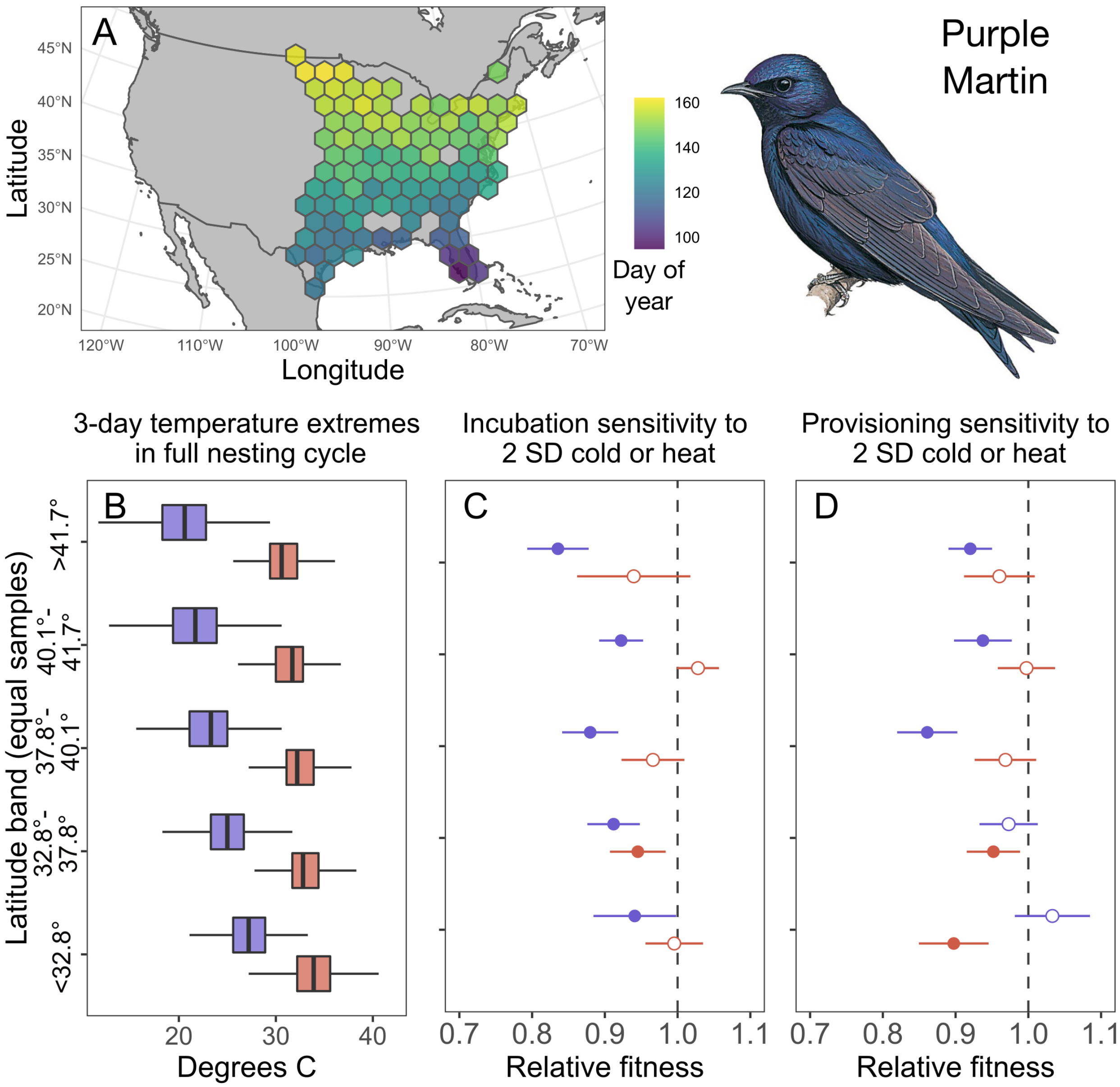

Figure S6: Breeding timing, temperature exposure, and extreme temperature sensitivity in purple martins. (A) Average date of first egg laying across the records included. (B) Distribution of the coldest and hottest 3-day high temperatures in the full nesting cycles in five latitude bands with an equal number of samples per band. The top of the panel is the northernmost band and bottom is the southernmost. Boxplots indicate median, IQR, and 1.5 times IQR for the coldest (blue) and hottest (red) 3-day period. (C and D) Predicted relative fitness for breeding attempts that experienced a 2 SD cold snap (blue) or 2 SD heatwave (red) during incubation (C) or provisioning (D) for each latitude band. Point estimates are derived from GAMs that control for spatial patterns, year, and date. Circles show point estimate, lines show 95 percent CI; open circles indicate no difference from mean reproductive success while closed circles indicate a significant effect of temperature on relative reproductive success. Illustration from Handbook of the Birds of the World by Hilary Bum, copyright Lynx Edicions.

Table S4: Point estimates for the expected relative fitness of purple martins during a two standard deviation cold snap or heatwave based on GAMs described in text. Breeding records are split into five latitude bands with an equal number of records per band; the southernmost band includes nests below 32.8 degrees latitude and is followed by 32.8 to 37.8 degrees, 37.8 to 40.1 degrees, 40.1 to 41.7 degrees, and finally nests above 41.7 degrees in the northernmost band. Numbers in brackets show 95-percent confidence intervals.

	Incubation cold snap	Incubation heatwave	Provisioning cold snap	Provisioning heatwave
Northernmost	0.84 [0.79, 0.88]	0.94 [0.86, 1.02]	0.92 [0.89, 0.95]	0.96 [0.91, 1.01]
North	0.92 [0.89, 0.95]	1.03 [1.00, 1.06]	0.94 [0.90, 0.98]	1.00 [0.96, 1.04]
Middle	0.88 [0.84, 0.92]	0.97 [0.92, 1.01]	0.86 [0.82, 0.90]	0.97 [0.93, 1.01]
South	0.91 [0.88, 0.95]	0.95 [0.91, 0.98]	0.97 [0.93, 1.01]	0.95 [0.92, 0.99]
Southernmost	0.94 [0.88, 1.00]	1.00 [0.96, 1.04]	1.03 [0.98, 1.08]	0.90 [0.85, 0.95]

Tree swallow sensitivity to cold snaps and heatwaves

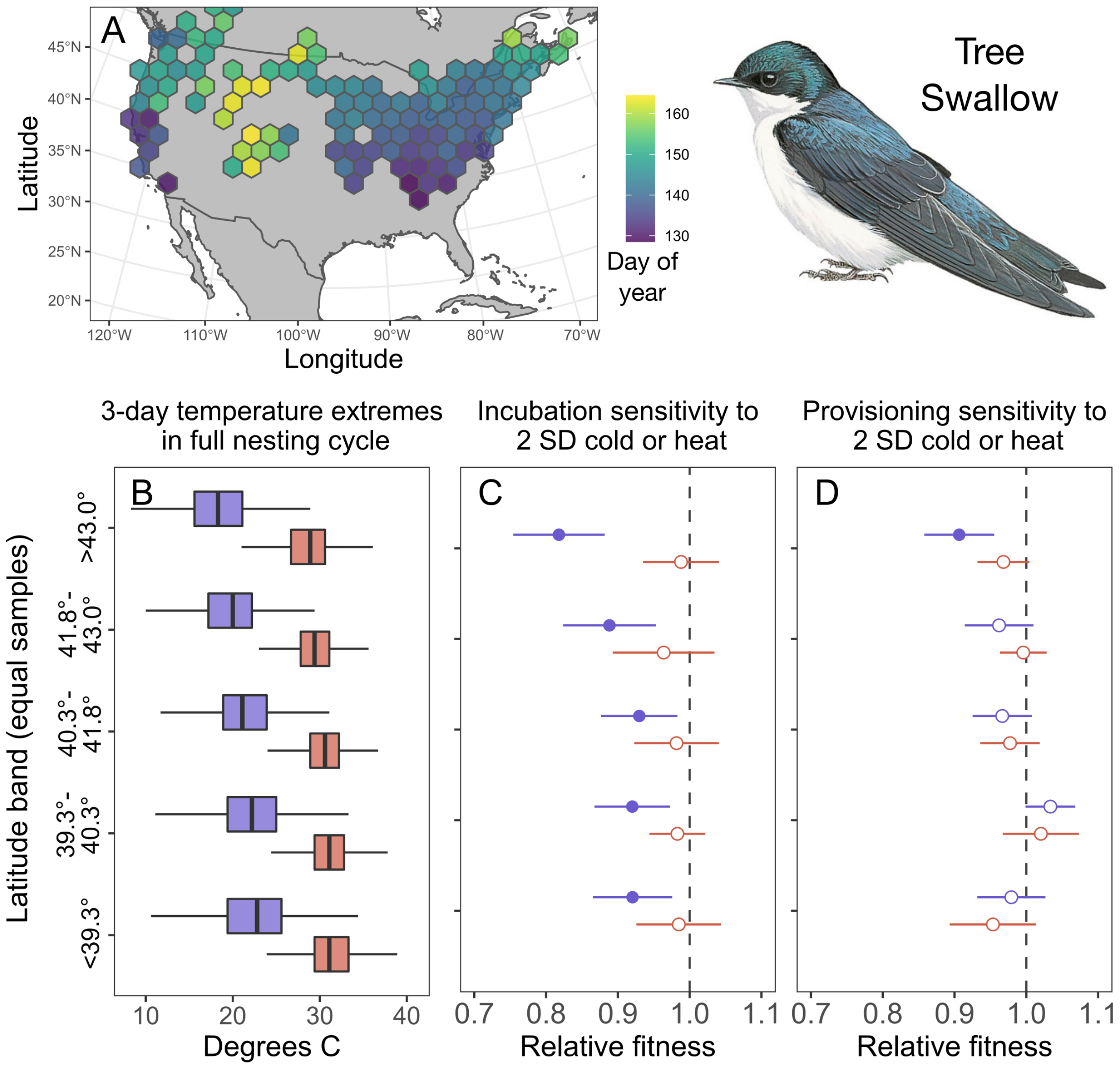

Figure S7: Breeding timing, temperature exposure, and extreme temperature sensitivity in tree swallows. (A) Average date of first egg laying across the records included. (B) Distribution of the coldest and hottest 3-day high temperatures in the full nesting cycles in five latitude bands with an equal number of samples per band. The top of the panel is the northernmost band and bottom is the southernmost. Boxplots indicate median, IQR, and 1.5 times IQR for the coldest (blue) and hottest (red) 3-day period. (C and D) Predicted relative fitness for breeding attempts that experienced a 2 SD cold snap (blue) or 2 SD heatwave (red) during incubation (C) or provisioning (D) for each latitude band. Point estimates are derived from GAMs that control for spatial patterns, year, and date. Circles show point estimate, lines show 95 percent CI; open circles indicate no difference from mean reproductive success while closed circles indicate a significant effect of temperature on relative reproductive success. Analysis by latitude band included only species in the eastern range, see methods for details. Illustration from Handbook of the Birds of the World by Hilary Bum, copyright Lynx Edicions.

Table S5: Point estimates for the expected relative fitness of tree swallows during a two standard deviation cold snap or heatwave based on GAMs described in text. Breeding records are split into five latitude bands with an equal number of records per band; the southernmost band includes nests below 39.3 degrees latitude and is followed by 39.3 to 40.3 degrees, 40.3 to 41.8 degrees, 41.8 to 43 degrees, and finally nests above 43 degrees in the northernmost band. Numbers in brackets show 95-percent confidence intervals.

	Incubation cold snap	Incubation heatwave	Provisioning cold snap	Provisioning heatwave
Northernmost	0.82 [0.75, 0.88]	0.99 [0.93, 1.04]	0.91 [0.86, 0.96]	0.97 [0.93, 1.00]
North	0.89 [0.82, 0.95]	0.96 [0.89, 1.03]	0.96 [0.91, 1.01]	1.00 [0.96, 1.03]
Middle	0.93 [0.88, 0.98]	0.98 [0.92, 1.04]	0.97 [0.93, 1.01]	0.98 [0.94, 1.02]
South	0.92 [0.87, 0.97]	0.98 [0.94, 1.02]	1.03 [1.00, 1.07]	1.02 [0.97, 1.07]
Southernmost	0.92 [0.87, 0.98]	0.98 [0.93, 1.04]	0.98 [0.93, 1.03]	0.95 [0.89, 1.01]
