# Supplementary material for: Inconsistent shifts in warming and temperature variability are linked to reduced avian fitness
Source: Nat Commun. 2023 Nov 16;14:7400. doi: 10.1038/s41467-023-43071-y (PMC10654519; doi:10.1038/s41467-023-43071-y)
Supplement: Supplementary file 3 — Reporting Summary [file 41467_2023_43071_MOESM3_ESM.pdf]

nature portfolio

Corresponding author(s): Conor C Taff

Last updated by author(s): 10/24/2023

Reporting Summary

Nature Portfolio wishes to improve the reproducibility of the work that we publish. This form provides structure for consistency and transparency in reporting. For further information on Nature Portfolio policies, see our Editorial Policies and the Editorial Policy Checklist.

Statistics

For all statistical analyses, confirm that the following items are present in the figure legend, table legend, main text, or Methods section.

n/a	Confirmed	
☐	☒	The exact sample size (*n*) for each experimental group/condition, given as a discrete number and unit of measurement
☐	☒	A statement on whether measurements were taken from distinct samples or whether the same sample was measured repeatedly
☐	☒	The statistical test(s) used AND whether they are one- or two-sided<br>*Only common tests should be described solely by name; describe more complex techniques in the Methods section.*
☐	☒	A description of all covariates tested
☐	☒	A description of any assumptions or corrections, such as tests of normality and adjustment for multiple comparisons
☐	☒	A full description of the statistical parameters including central tendency (e.g. means) or other basic estimates (e.g. regression coefficient) AND variation (e.g. standard deviation) or associated estimates of uncertainty (e.g. confidence intervals)
☐	☒	For null hypothesis testing, the test statistic (e.g. *F*, *t*, *r*) with confidence intervals, effect sizes, degrees of freedom and *P* value noted<br>*Give P values as exact values whenever suitable.*
☐	☒	For Bayesian analysis, information on the choice of priors and Markov chain Monte Carlo settings
☐	☒	For hierarchical and complex designs, identification of the appropriate level for tests and full reporting of outcomes
☐	☒	Estimates of effect sizes (e.g. Cohen's *d*, Pearson's *r*), indicating how they were calculated

Our web collection on statistics for biologists contains articles on many of the points above.

Software and code

Policy information about availability of computer code

Data collection	No new data were collected.
Data analysis	All data processing, analyses, and figures were created in R version 4.0.2. We used the following R packages in our analysis: tidyverse v1.3.0; rnoaa v1.3.6.94; sf v0.9-8; rnaturalearth v0.1.0; ggpubr v0.4.0; data.table v1.14.2; weathercan v0.5.0; elevatr v0.3.4; mgcv v1.8-31; and dplyr v1.0.8. A complete set of annotated code to reproduce the full analysis, manuscript, and supplemental materials is permanently archived on Zenodo (DOI: 10.5281/zenodo.6011738).

For manuscripts utilizing custom algorithms or software that are central to the research but not yet described in published literature, software must be made available to editors and reviewers. We strongly encourage code deposition in a community repository (e.g. GitHub). See the Nature Portfolio guidelines for submitting code & software for further information.

Data

Policy information about availability of data

All manuscripts must include a data availability statement. This statement should provide the following information, where applicable:

- Accession codes, unique identifiers, or web links for publicly available datasets
- A description of any restrictions on data availability
- For clinical datasets or third party data, please ensure that the statement adheres to our policy

The nesting data used in this study were obtained from three citizen science databases and can be retrieved from each of them. Both the NestWatch program and

Project Nestwatch have data access portals (https://nestwatch.org/nw/public/export and https://www.birdscanada.org/bird-science/project-nestwatch). Access to data can be obtained by submitting a request and agreeing to data use policies. Data from Project Martwinwatch are publicly archived on Dryad (doi:10.5061/dryad.msbcc2fwq).
Historical temperature data were accessed from the Berkeley Earth project (www.berkeleyearth.org/data/) or from meteorological station records maintained by NOAA in the United States (https://www.ncei.noaa.gov/cdo-web/) and by ECCC in Canada (https://climate.weather.gc.ca/historical_data/search_historic_data_e.html).

Research involving human participants, their data, or biological material

Policy information about studies with human participants or human data. See also policy information about sex, gender (identity/presentation), and sexual orientation and race, ethnicity and racism.

Reporting on sex and gender	NA
Reporting on race, ethnicity, or other socially relevant groupings	NA
Population characteristics	NA
Recruitment	NA
Ethics oversight	NA

Note that full information on the approval of the study protocol must also be provided in the manuscript.

Field-specific reporting

Please select the one below that is the best fit for your research. If you are not sure, read the appropriate sections before making your selection.

☐ Life sciences ☐ Behavioural & social sciences ☒ Ecological, evolutionary & environmental sciences

For a reference copy of the document with all sections, see nature.com/documents/nr-reporting-summary-flat.pdf

Ecological, evolutionary & environmental sciences study design

All studies must disclose on these points even when the disclosure is negative.

Study description	We combined >300,000 community science breeding records with data on historical temperature to understand how exposure to extreme climatic events has changed for 24 North American songbirds during breeding. We used the breeding records to ask whether exposure to a cold-snap or heat-wave during breeding resulted in reduced reproductive success for each of these species.
Research sample	We included all breeding records available from three community science projects: Nestwatch, Project Nestwatch, and Project MartinWatch. Using these databases we included all species that had sufficient sample sizes for analysis, which resulted in analysis of 24 species. The sample is meant to represent range wide breeding data for the included species.
Sampling strategy	No pre-determined sample size calculation was used. Instead, we used all available data from public archives. This resulted in >300,000 breeding records from 24 common species, which was ample to analyze the main questions we addressed. To arrive at this final number we employed a variety of inclusion criteria involving filtering for missing data, probable data entry errors, and availability of temperature data.
Data collection	We did not collect any new data for this study. We used publicly available databases. Data were gathered by Conor Taff, who contacted Nestwatch and Program Nestwatch for bulk downloads and accessed MartwinWatch data from Dryad (doi: 10.5061/dryad.msbcc2fwq).
Timing and spatial scale	We used breeding records reported from 1995-2020. This corresponded with the start of the community science databases and very few records were available from earlier than 1995. The spatial extent of the data covered the United States and Canada.
Data exclusions	We only excluded records that did not include the necessary data for analysis. Because we used community science data, records could be insufficient in a variety of ways (e.g., incomplete, impossible values suggesting data entry errors, etc). We fully describe these exclusion criteria in the manuscript. All exclusion was based only on data completeness and was developed and performed before the statistical analyses were carried out.
Reproducibility	We did not collect any new data or perform any new experiments for this analysis. We ensured reproducibility of the analysis by documenting the complete coding and analysis pipeline and publicly archiving this material with our submission.
Randomization	Randomization was not relevant to our study because we examined a large database of naturally occurring nest records. We did not collect any new data or apply any experimental treatments.

Blinding | Blinding was not relevant because we did not apply any treatments or produce any new measurements of records.

Did the study involve field work? ☐ Yes ☒ No

Reporting for specific materials, systems and methods

We require information from authors about some types of materials, experimental systems and methods used in many studies. Here, indicate whether each material, system or method listed is relevant to your study. If you are not sure if a list item applies to your research, read the appropriate section before selecting a response.

Materials & experimental systems

n/a	Involved in the study	
☒	☐	Antibodies
☒	☐	Eukaryotic cell lines
☒	☐	Palaeontology and archaeology
☐	☒	Animals and other organisms
☒	☐	Clinical data
☒	☐	Dual use research of concern
☒	☐	Plants

Methods

n/a	Involved in the study	
☒	☐	ChIP-seq
☒	☐	Flow cytometry
☒	☐	MRI-based neuroimaging

Animals and other research organisms

Policy information about studies involving animals; ARRIVE guidelines recommended for reporting animal research, and Sex and Gender in Research

Laboratory animals	NA
Wild animals	Data from wild animals were used but we did not collect any new data. All data was accessed from community science databases.
Reporting on sex	NA
Field-collected samples	NA
Ethics oversight	NA

Note that full information on the approval of the study protocol must also be provided in the manuscript.
